# Temporal Relationship between Vitamin D Status and Parathyroid Hormone in the United States

**DOI:** 10.1371/journal.pone.0118108

**Published:** 2015-03-04

**Authors:** Martin H. Kroll, Caixia Bi, Carl C. Garber, Harvey W. Kaufman, Dungang Liu, Anne Caston-Balderrama, Ke Zhang, Nigel Clarke, Minge Xie, Richard E. Reitz, Stephen C. Suffin, Michael F. Holick

**Affiliations:** 1 Quest Diagnostics, 3 Giralda Farms, Madison, NJ, United States of America; 2 Quest Diagnostics Nichols Institute, 33608 Ortega Highway, San Juan Capistrano, CA, United States of America; 3 Office of Statistical Consulting, Department of Statistics and Biostatistics, Rutgers University, 110 Frelinghuysen Road, Piscataway, NJ, United States of America; 4 Department of Biostatistics, Yale University School of Public Health, 300 George Street, New Haven, CT, United States of America; 5 Department of Medicine, Physiology and Biophysics at Boston University School of Medicine, Boston University School of Medicine, 88 East Newton, Boston, MA, 02118, United States of America; University Medical Center Groningen and University of Groningen, NETHERLANDS

## Abstract

**Background:**

Interpretation of parathyroid hormone (iPTH) requires knowledge of vitamin D status that is influenced by season.

**Objective:**

Characterize the temporal relationship between 25-hydroxyvitamin D_3_ levels [25(OH)D_3_] and intact iPTH for several seasons, by gender and latitude in the U.S. and relate 25-hydrovitamin D_2_ [25(OH)D_2_] levels with PTH levels and total 25(OH)D levels.

**Method:**

We retrospectively determined population weekly-mean concentrations of unpaired [25(OH)D_2_ and 25(OH)D_3_] and iPTH using 3.8 million laboratory results of adults. The 25(OH)D_3_ and iPTH distributions were normalized and the means fit with a sinusoidal function for both gender and latitudes: North >40, Central 32–40 and South <32 degrees. We analyzed PTH and total 25(OH)D separately in samples with detectable 25(OH)D_2_ (≥4 ng/mL).

**Findings:**

Seasonal variation was observed for all genders and latitudes. 25(OH)D_3_ peaks occurred in September and troughs in March. iPTH levels showed an inverted pattern of peaks and troughs relative to 25(OH)D_3_, with a delay of 4 weeks. Vitamin D deficiency and insufficiency was common (33% <20 ng/mL; 60% <30 ng/mL) as was elevated iPTH levels (33%>65 pg/mL). The percentage of patients deficient in 25(OH)D_3_ seasonally varied from 21% to 48% and the percentage with elevated iPTH reciprocally varied from 28% to 38%. Patients with detectable 25(OH)D_2_ had higher PTH levels and 57% of the samples with a total 25(OH)D > 50 ng/mL had detectable 25(OH)D_2_.

**Interpretation:**

25(OH)D_3_ and iPTH levels vary in a sinusoidal pattern throughout the year, even in vitamin D_2_ treated patients; 25(OH)D_3_, being higher in the summer and lower in the winter months, with iPTH showing the reverse pattern. A large percentage of the tested population showed vitamin D deficiency and secondary hyperparathyroidism. These observations held across three latitudinal regions, both genders, multiple-years, and in the presence or absence of detectable 25(OH)D_2_, and thus are applicable for patient care.

## Introduction

Parathyroid hormone (PTH) concentrations are known to have a reciprocal seasonal relationship with 25(OH)D in studies lasting up to a year [1–5]. Chapuy et al showed that after the sixth decade there was a decline in 25(OH)D_3_ and increase in parathyroid hormone [1]. Lips et al showed that parathyroid hormone was highest in the winter, when total 25(OH)D [25(OH)D_2_ and 25(OH)D_3_] were at their lowest values, suggesting a secondary phenomenon, in 124 patients [2]. Krall et al and Tangpricha et al demonstrated an inverse relationship between parathyroid hormone and 25(OH)D_3_ over one season [3,4]. The inverse relationship was present when vitamin D intake was less than 220 IU/day, but not observed when the intake exceeded this amount [3]. Because deficiency in vitamin D and elevation of parathyroid hormone is believed to have an impact on bone fragility and other organ systems, their relationship is important [6,7]. The temporal relationship provides information on the impact of time of testing and the dynamic interactions between the two analytes, but the previous studies have been limited by short duration, constraints on sample size, geographic scope, and population characteristics such as age and gender. Studies limited to one year in duration are insufficient to characterize the recurrent temporal relationship. To avoid these limitations, we investigated the seasonal variation of 25(OH)D and iPTH over a multi-year period using data drawn from a large clinical data base comprising results from more than 3.8 million patients from throughout the continental United States who sought laboratory testing for 25(OH)D or iPTH.

## Methods

### Study population

The study included 2,274,884 25(OH)D_3_ results and 1,529,289 iPTH results (in the Quest Diagnostics database), performed as routine care, for patients of both genders, spanning the ages of 20 to 99 years, living within the continental United States, from January 2007 through December 2009 in the Quest Diagnostics database. 7% of patients had a diagnosis of vitamin D deficiency (ICD-9 code 268.9) were included. In addition, we examined matched values of iPTH with 25(OH)D_total_, 25(OH)D_3_ and 25(OH)D_2_, subdividing them into four groups: 25(OH)D_3_ for all subjects, 25(OH)D_3_ for subjects without detectable for 25(OH)D_2_ (<4 ng/mL25(OH)D_total_ for subjects with detectable for 25(OH)D_2_ (≥4 ng/mL), and 25(OH)D_total_ ≥ 50 ng/mL. The rationale for the subgroups is that patients with detectable 25(OH)D_2_ are likely to have been treated for vitamin D deficiency with the only FDA approved pharmaceutical, vitamin D_2_. For iPTH we excluded values greater than 195 pg/mL because they are three-fold higher than the upper limit of normal. The total number excluded based on this criteria was 87,744 representing only 5% of the samples. In addition we excluded those samples associated with ICD-9 diagnostic codes indicating chronic kidney disease (codes: 285.21, 403.00, 403.01, 403.10, 403.11, 403.90, 403.91, 404.00, 404.01, 404.02, 404.03, 404.10, 404.11, 404.12, 404.13, 404.90, 404.91, 404.92, 404.93, 585.1, 585.2, 585.3, 585.4, 585.5,585.9). The total number of excluded values was 464,177. Further checks were done using the eGFR and only 5% of patients with CKD lack an appropriate code and only 13% of patients without a CKD code had an eGFR indicating stage 3 or higher, thus the number missed is less than 1%.

To estimate the effect of latitude, we characterized into 3 regions based on the ZIP code of the patient’s home address (http://federalgovernmentzipcodes.us/ updated 3/9/2011),: North (>40 degrees), Center (32–40 degrees), and South (<32 degrees).

This study was found to be exempt for human studies research by the Western Institutional Review Board. All data were de-identified prior to analysis.

### Laboratory Analysis

Total 25(OH)D measurements {25(OH)D_2_ and 25(OH)D_3_} were performed using liquid chromatography—tandem mass spectrometry (ThermoFisher, San Jose, CA). iPTH measurements were performed using a chemoluminescence-based assay (Siemens Immulite 2000, Deerfield, IL) over the course of the study. The reference ranges for the course of the study were 30 to 100 ng/mL for total 25(OH)D and 10 to 65 pg/mL for iPTH. The assays were standardized and performed by the same methods in each laboratory (34 in total) and year-to-year.

### Statistical analysis

We analyzed unmatched and paired 25(OH)D_3_ and PTH data. Also, we analyzed subgroups based on detectability of 25(OH)D_2_ and 25(OH)D_total_ > 50 ng/mL.

We determined the weekly mean 25(OH)D_3_ and iPTH concentrations for the overall population, gender and region, normalizing results using a Box-Cox power estimator function (R, version 2.13.0, The R Foundation for Statistical Computing, Auckland, New Zealand). The sine curve was fitted in R using the “nls” function (nonlinear least-square fit) as follows: $nls\left(y\sim b_{0}+A∙\sin\left(\frac{2∙\pi }{T}\left(x+\varphi \right)\right),\;start\;=\;list\left(b_{0}\;=\;1,A\;=\;1,\varphi \;=\;1\right)\right)$, where b0 is the intercept, A is the amplitude of the Sine wave, T is the cycle (52 weeks in this case) and φ is the offset of the cycle. The transformation estimator was 0.5 for 25(OH)D_3_ and 0.3 for iPTH, then fitted values were back-transformed. The seasonality model included both an intercept (ß_0_) and a periodic term. The periodic terms of *t* (time in weeks) and cycle time, *T* (52 weeks), describe the seasonal variations of the 25(OH)D_3_ and iPTH.

The model is:

$$y\;=\;b_{0}+A∙\sin\left(\frac{2∙\pi }{T}\left(x+\varphi \right)\right)+\varepsilon$$ (equation 1)

Where:

*y = Polynomial term [for 25(OH)D*_*3*_ *] + Seasonal term + measurement uncertainty*

*A* = the amplitude/height of the sine wave

φ = the phase angle, representing the difference from time zero and serves as a reference to the periods of sunlight exposure.

The standard errors of parameters for each group (gender, region, and delay) were used to calculate the confidence interval for each parameter and test for the significance of differences. The agreement between the data and the fitted model had a mean squared error of 3.6 (7%) for the PTH seasonal model and 2 (8%) for the 25(OH)D_3_ seasonal model.

## Results

### Patient Characteristics

Table 1 provides information on age, gender, and latitude regions for 25(OH)D_3_ and iPTH.

**Table 1:** Characteristics of Populations Tested for 25-Hydroxyvitamin D_3_, Intact PTH (iPTH).

		Number	Mean (SD) Age, y
**25-hydroxyvitamin D**_**3**_	Women	1,692,905	59.6 (16.0)
	Men	581,979	60.9 (16.0)
	Total	2,274,884	59.9 (16.0)
	Northern	1,573,144	59.4 (16.2)
	Central	600,598	60.8 (15.6)
	Southern	101,142	63.1 (15.9)
**iPTH**	Women	1,043,091	62.5 (15.2)
	Men	486,198	63.2 (15.6)
	Total	1,529,289	62.7 (15.3)
	Northern	609,176	62.7 (15.4)
	Central	240,687	62.2 (15.2)
	Southern	679,426	62.9 (15.3)

### 25(OH)D_3_ seasonal variation and distribution

**Fig 1:**
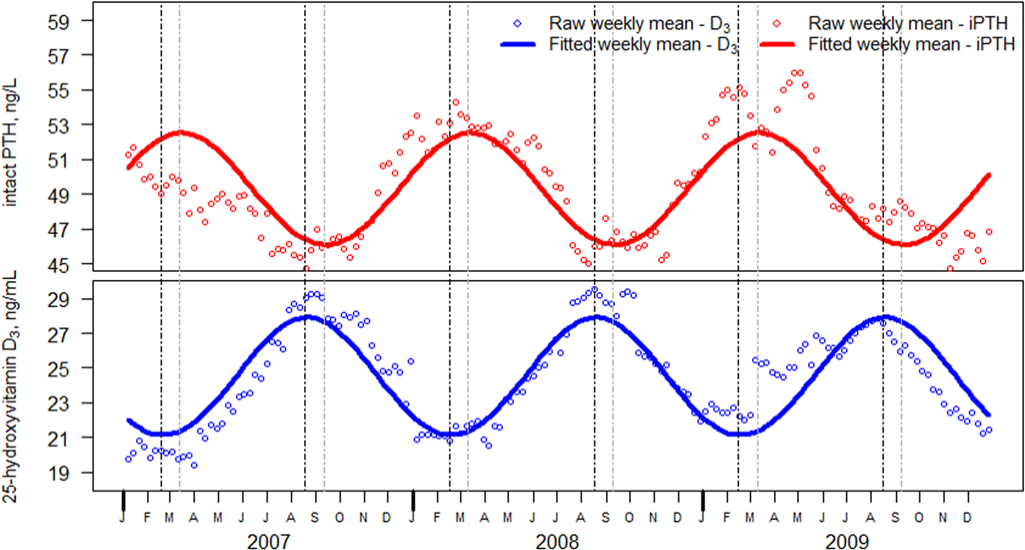
Seasonal Variation of 25-Hydroxyvitamin D_3_ (bottom panel) and Intact PTH (iPTH) (top panel) Weekly Mean Values. The maximum seasonal variation of 25(OH)D_3_ (peak to trough) was 6.8 ng/mL, reaching its trough in the 8^th^ week (early March) of each year and its peak in the 34^th^ week (early September). Peak iPTH values occurred at week 12 (early April) and trough values at week 37 (late September), a pattern that is roughly reciprocal to that of 25(OH)D_3_, but lags by 3.5 weeks. Individual points represent the mean of the normalized distribution for each week. The solid lines represent the fit. Dark vertical dashed lines represent 25-hydroxyvitamin D_3_ peaks and troughs, and light vertical dashed lines represent the iPTH peaks and troughs. To convert 25-hydroxyvitamin D_3_ from ng/mL to nmol/L, multiply by 2.496 (rounded as 2.5).

**Fig 2:**
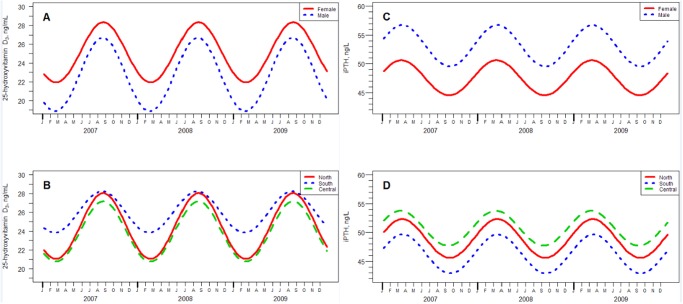
Seasonal Variation in Weekly Mean Values of 25-Hydroxyvitamin D_3_ (panels A and B) and Intact PTH (iPTH; panels C and D) by Gender and Region. A. Women show higher average values for 25(OH)D_3_ than men. B. North and Central regions show similar average values for 25(OH)D_3_ but the South region shows higher average values in winter, even though all three regions show similar values in summer. C. Men show higher average values than women for iPTH. D. The Central region shows the highest average values for iPTH, followed by the Northern region and then the South region.

Both 25(OH)D_3_ and iPTH showed a seasonal variation, iPTH reciprocal to that of 25(OH)D_3_, for both genders and all regions (Figs. 1 and 2). The average number of samples per week for 25(OH)D_3_ was 14,583 (standard deviation of 3949), and the average standard error for the weekly mean was 0.05 ng/mL. The maximum seasonal variation of 25(OH)D_3_ (peak to trough) was 6.8 ng/mL. The model indicates that 25(OH)D_3_ reached its trough in the 8^th^ week (end of February to early March) and its peak in the 34^th^ week (end of August to early September), with a period between the peaks and troughs 26 weeks.

**Fig 3:**
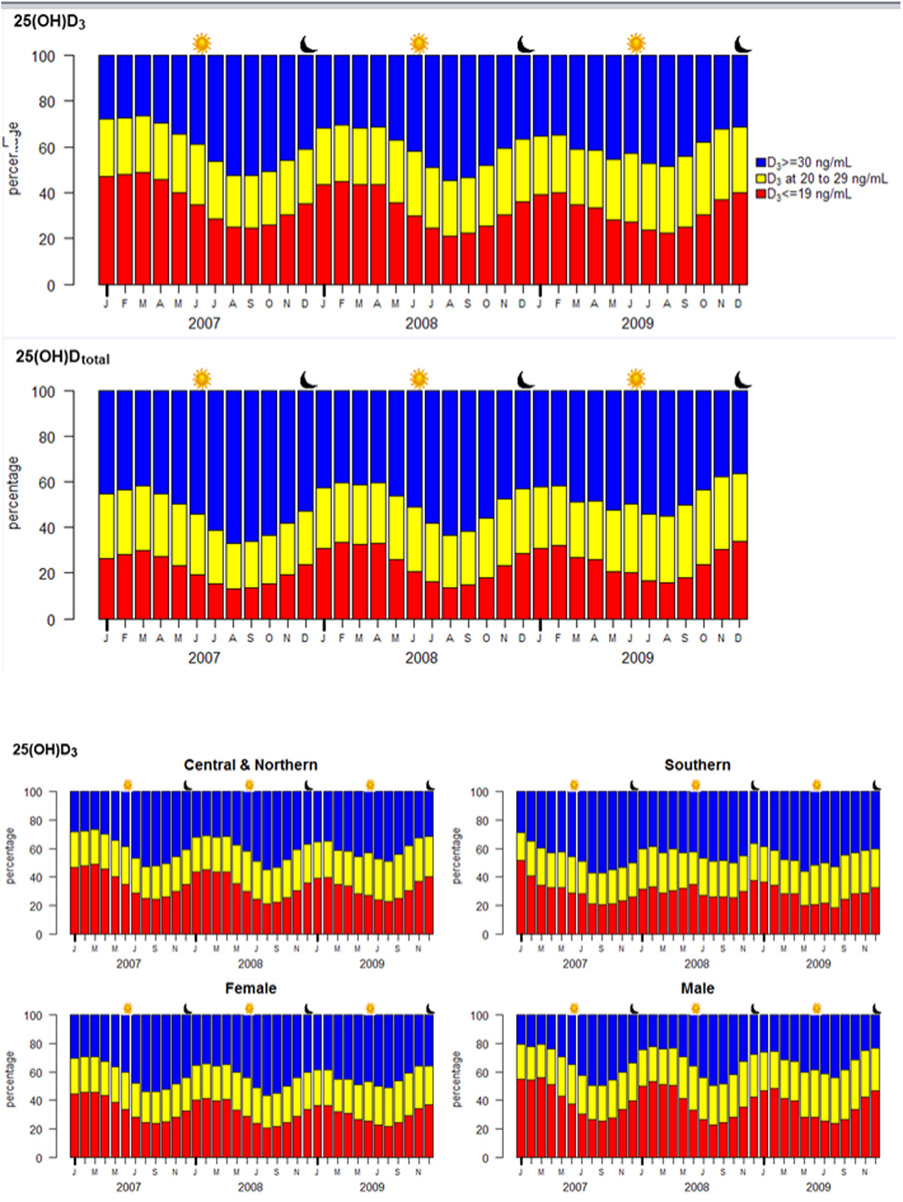
Percentages of patients with 25(OH)D_3_ deficiency (<20 ng/mL), insufficiency (20–29 ng/mL), and sufficiency (≥30 ng/mL), by month. The percentiles are categorized by month. The upper portion (blue) of each month shows the percentage of patients with sufficient 25(OH)D_3_; the central portion (yellow), insufficient but not deficient; and the lower portion (red), deficient. The percentage of patients considered deficient or insufficient depends on the season, lower in summer (approximately 50%) and higher in winter (approximately 70%). The sun symbol indicates the summer solstice and the crescent moon symbol, the winter solstice. The central panel shows the seasonal similarity of total 25(OH)D with that of 25(OH)D2. The lower panel shows the rhythmic pattern across regions and gender. The Central and Northern regions were combined because they were similar. Their trough to peak difference is greater than that for the Southern region. Women and men percentages show similar patterns, except that the trough to peak difference is greater for men than women. The sun symbol indicates the summer solstice and the crescent moon, the winter solstice.

Men had a greater peak-trough difference than women (7.8 versus 6.4 ng/mL) (p<0.01). Average values for men and women remained below 30 ng/mL and men’s values fell below 20 ng/mL. 25(OH)D_3_ levels were 2.4 ng/mL lower in men than in women (P < 0.001). Patients in the North had the greatest peak-trough difference (7.0 ng/mL) and those living in South had the least (4.4 ng/mL) (Fig. 2B). Fig. 3 shows the cutoffs for insufficiency and sufficiency respectively [7].

### iPTH seasonal variation and distribution

Seasonal variation was observed for iPTH overall, for both genders, and for all regions (Fig. 1). The weekly iPTH mean (9803 samples per week with a standard deviation of 1547) had a standard error of 0.07 pg/mL. Peak values occurred at the 12^th^ week (March) and trough values at the 37^th^ week (September), reciprocal and one month later compared to 25(OH)D_3_.

iPTH followed a sinusoidal pattern for both genders and all regions (Fig. 2C and D), with identical amplitudes, but men demonstrating higher values than women (Table 2). The values for iPTH were lowest in the South and highest in the Center, (p<0.01). The lag time between 25(OH)D_3_ and iPTH differed for the central and southern regions (p<0.05), but not for the central and northern or northern and southern regions (p>0.05).

**Table 2:** Time difference between peak 25(OH)D_3_ and trough iPTH (weeks) for Region and Gender Variation.

	25-hydroxyvitamin D_3_			iPTH			
	Average Baseline ng/mL*	Amplitude of Peak to Trough difference ng/mL	Week of Peak φ adjusted**	Average Baseline, pg/mL	Amplitude of Peak to Trough difference pg/mL	Week of Peak φ adjusted**	Difference in peak between D3 and trough PTH, week
**Northern**	24.4	7.0	33.9	48.87	6.71	11.5	3.6
**Central**	23.9	6.4	34.0	50.67	6.09	10.6	2.7
**Southern**	26.0	4.4	33.3	46.19	6.76	11.8	4.6
**Women**	25.0	6.4	34.0	47.51	6.08	11.1	3.1
**Men**	22.6	7.8	33.6	53.06	7.19	11.5	3.9
**Total**	24.4	6.8	33.8	49.23	6.42	11.2	3.4

*To convert 25-hydroxyvitamin D_3_ to nanomoles per liter, multiply by 2.496.

**The standard errors for the Phase Angles for 25(OH)D and iPTH are 0.4 and 0.5, respectively. The Phase Angle represents the lag time where the sine function has a value of time = 0. The sine curves for 25(OH)D_3_ and iPTH differ by 26 weeks and their differences in their phase angles.

### Percentage of patients deemed deficient or insufficient in 25(OH)D_3_

Fig. 3 shows the sinusoidal pattern of 25(OH)D_3_ (in months) of the percentage of patients stratified by cutoffs of 20 ng/mL or 30 ng/mL 25(OH)D_3_. The upper panel shows the percentage of patients with 25(OH)D_3_ values greater than 30 ng/mL (blue), while the lower portion of the panel shows the percentage of patients with values less than 20 ng/mL (red). During the months when 25(OH)D_3_ values were at their lowest, more than 70% of patients had values less than 30 ng/mL and more than 40% had values less than 20 ng/mL. The temporal pattern for total 25(OH)D is essentially the same as that for 25(OH)D_3_ (middle panel). The sinusoidal patterns remain the same for gender and region (Fig. 3). At the lowest values for 25(OH)D_3_ during the year approximately 60% of subjects in the southern region and 70% of the central and northern region subjects would be considered deficient or insufficient. Men showed 80% below 30 ng/mL and women, 70%.

### Percentage of patients with elevated iPTH

**Fig 4:**
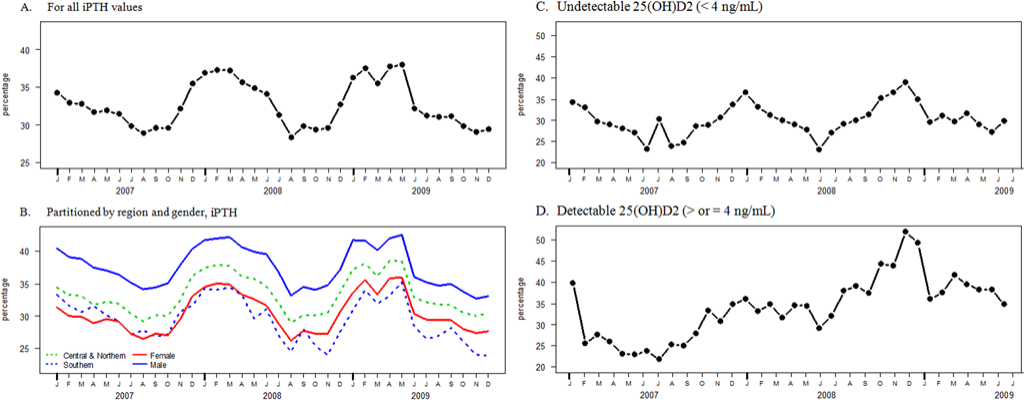
Percentage of patients with elevated iPTH concentrations (>65 pg/mL), by month. A. All groups. B. The percentage of subjects with iPTH greater than the upper limit of the reference interval (>65 pg/mL) reaches its trough in late summer and its peak in late winter. C. By region and gender. D. Patients with undetectable 25(OH)D_2_ E. All patients with detectable 25(OH)D_2_, even though the sinusoidal pattern is somewhat diminished, it still fit a sinusoidal function (null hypothesis p<0.001). The seasonal relationship holds across region, gender and presence or absence of detectable 25(OH)D_2_. The regions demonstrate similar patterns, but the percentage of subjects with iPTH greater than the reference interval was greater for the central and northern regions than the southern region and greater for men than women.

The population normalized means for iPTH differ for men and women (47.5 pg/mL for women (80^th^ percentile of normal population) and 53.1 pg/mL (86^th^ percentile) for men (p<0.01). Fig. 4A shows the percentage of patients with iPTH greater than 65 pg/mL; on average, 33% of iPTH results exceeded the upper limit of the reference interval. Fig. 4B shows the consistent seasonal trends of iPTH exceeding the upper limit by region (north and central combined) and gender.

### Examination of 25(OH)D_2_ and 25(OH)D_3_ subsets

A total of 56,713 subjects had paired iPTH and 25(OH)D values. Of these, 69.4% (39,385) had undetectable and 30.6% (17,328) had detectable 25(OH)D_2_, 10.8% (6,113) had 25(OH)D_total_>50 ng/mL and 5.2% (2,925) had 25(OH)D_3_>50 ng/mL.

**Fig 5:**
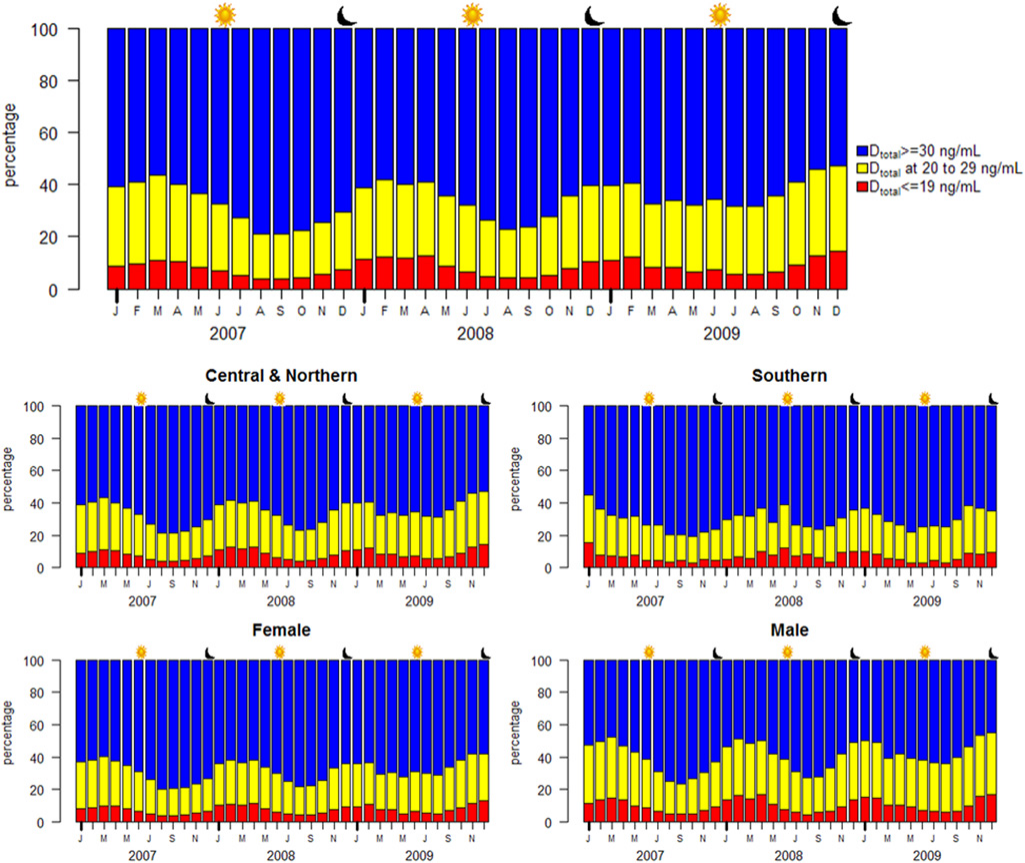
Categorical percentage of patients for total 25(OH)D for all patients who had detectable 25(OH)D_2_. The percentiles are categorized by month. The upper portion (blue) of each month shows the percentage of patients with sufficient 25(OH)D_3_; the central portion (yellow), insufficient but not deficient; and the lower portion (red), deficient.

Subjects with detectable 25(OH)D_2_ and 25(OH)D_total_>50 ng/mL retained the seasonal pattern with similar amplitude and phase (fig. 5). The normalized mean value for iPTH was 50.2 ng/L for those with detectable 25(OH)D_2_, 47.4 ng/L for those with undetectable 25(OH)D_2_ and 38.2 ng/L for those with 25(OH)D_total_ >50 ng/mL (Table 3). Table 3 shows that patients with measurable 25(OH)D_2_ had a greater percentage with iPTH>65 ng/L (P<0.001) than those without measurable 25(OH)D_2_ even though they had greater total 25(OH)D than those with no detectable 25(OH)D_2_ they did not lose their seasonal relationship (the data still significantly fits to a sinusoidal curve with the probability of the null hypothesis <0.001) (fig. 4C and D). Subjects with detectable 25(OH)D_2_ had nearly equal means of 25(OH)D_3_ and 25(OH)D_2_ as well as more 25(OH)D_total_ than the group with undetectable 25(OH)D2 (p<0.001). In subjects with detectable 39% of the total 25(OH)D was composed of 25(OH)D_2_. In subjects with 25(OH)Dtotal>50 ng/mL 19% of the total 25(OH)D was composed of 25(OH)D_2_.

**Table 3:** Normalized mean values of 25(OH)D and iPTH for three subgroups.

Normalized Mean	iPTH	25(OH)D_2_	25(OH)D_3_	25(OH)D_total_	% iPTH>65 pg/mL
	ng/L	ng/mL	ng/mL	ng/mL	
25(OH)D _total_>50 ng/mL	38.2	11.4	35.6	59.1	20%
25(OH)D2> = 4 ng/mL *	50.2	14.6	16.0	37.1	34%
25(OH)D2<4 ng/mL *	47.4		27.2	27.2	30%

* The values for iPTH and 25(OH)D between detectable and undetectable 25(OH)D_2_ are significantly different, p<0.001.

Of patients with total 25(OH)D>50 ng/mL, 2678 (57%) had detectable 25(OH)D_2_, and 2044 did not (Table 4). Subjects with 25(OH)D_total_ > 50 ng/mL in the south region were more likely to have undetectable than detectable 25(OH)D_2_ (P<0.0005) (Table 4).

**Table 4:** 25(OH)D_2_ status amongst patients with 25(OH)D_total_ > 50 ng/mL.

Dtotal > 50 ng/mL	N	%	North %	Central %	South %
D_2_ <4 ng/mL	2044	41%	73%	15%	12%
D_2_ > = 4 ng/mL	2678	59%	76%	15%	9%

P<0.0005 for the percentages for the southern region differentiated by detectable or not 25(OH)D_2_.

## Discussion

We have shown that both 25(OH)D_3_ and iPTH demonstrate consistent inversely proportional seasonal sinusoidal patterns across geographic regions and gender, and the peaks and troughs lag behind the times of the greatest or least amounts of daylight. For 25(OH)D_3_, the peak occurs during the 34^th^ week, early September, and the trough occurs during the ninth week, early March. For iPTH, its trough occurs 3.5 weeks after the 25(OH)D_3_ peak and its peak 3.5 weeks after the 25(OH)D_3_ trough. Men showed lower values of 25(OH)D_3_ and higher values of iPTH than women. The North and Center were merged, because their pattern and average values were similar, and they showed lower concentrations of 25(OH)D_3_ and higher concentrations of iPTH than the South during winter. This study of nearly 2.3 million 25(OH)D_3_ and 1.5 million iPTH test results represents the largest aggregation of data used to characterize a population over expanded geography and multiple years for these analytes and characterize this recurring temporal pattern.

Prior studies have shown that 25(OH)D followed a sinusoidal pattern over a year and that parathyroid hormone was inversely related in a limited number of subjects [8–13]. Additionally, the inverse relationship between 25(OH)D_3_ and parathyroid hormone during winter was associated with an increase in fractures [14]. These prior studies suggest that the lack of adequate sunlight exposure and the inability of sunlight to produce vitamin D during winter leads to increased parathyroid hormone concentrations, but their limited sample size and duration detract from the consistency of their observations. Our large sample size, normalized distributions, inclusion of both genders and multiple latitudinal regions demonstrate that these observations are reproducible and compatible with the reciprocal relationship between vitamin D and iPTH [14].

We found that men had a lower mean 25(OH)D_3_ and higher mean iPTH concentrations than women, consistent with more recent findings in the NHANES studies [15,16].

Our observations show that the central and northern regions experience the same seasonal variation, but the south region experiences a dampened response, which may relate to longer duration for vitamin D production in the south [17,18].

The lag time between vitamin D and iPTH extremes may relate to an accumulative effect of sunlight exposure over the summer months and the relatively long half-life of circulating 25(OH)D [19]. Vitamin D enters body fat and is presumably slowly released back into the circulation, thus, only a small fraction is converted to 25(OH)D, resulting in a slow but steady rise in serum 25(OH)D [20]. Once made a small fraction of 25(OH)D is then converted in the kidneys to 1,25(OH)_2_D.

Vitamin D deficiency results in a decrease in intestinal calcium absorption, resulting in a transient decline in ionized calcium concentrations in the serum [21]. The calcium sensor in the parathyroid gland detects the decrease, resulting in an increase in the production and secretion of PTH. The continued stimulation of the parathyroid glands results in a gradual increase in PTH levels that are sustained until there is improvement in serum 25(OH)D levels, when it gradually returns to baseline.

25(OH)D concentrations do not reach their lowest values until at least two months following the winter solstice, again reflecting the cumulative effect of decreased or absent cutaneous vitamin D_3_ synthesis during the winter months [9,22]. Values for 25(OH)D may be more pertinent when they are measured near their nadir, while increased values of iPTH at their apex may be more a reflection of a decrease in vitamin D status rather than parathyroid disease.

The percentage of patients considered vitamin D deficient [25(OH)D_3_ <20 ng/mL] broadly ranged from a trough of approximately 20% in August through September, to 50% in January through March (Fig. 3), but our observed 35% average deficiency agreed with recent NHANES studies [19,23,24]. The amplitude of 6.8 ng/dL represents 68% of the range between sufficiency and insufficiency. Other studies have described the seasonal relationship of 25(OH)D and iPTH, consistent with ours [25–30]. These results provide guidance for the interpretation of results based on the season and current guidelines may need to take the seasonality into consideration.

Patients whose specimens demonstrate detectable 25(OH)D_2_ (values ≥ 4 ng/mL) most likely have been treated with pharmaceutical doses of vitamin D_2_, probably because they were diagnosed as being vitamin D deficient or insufficient. Patients do have access to over-the-counter sources of vitamin D, but most of these are vitamin D_3_. Thus, those patients who had detectable 25(OH)D_2_ were most probably taking the pharmaceutical form of vitamin D_2_. In spite of more than sufficient total 25(OH)D, these patients did not lose their seasonal response for 25(OH)D or iPTH (figs. 4 and 5). Thus, being treated with vitamin D_2_ does not appear to affect serum 25(OH)D_3_, as has been previously reported [31]. Patients with detectable 25(OH)D_2_ had higher median iPTH than those with undetectable amounts, even though their total 25(OH)D was higher (Table 3). This could be due to patients who were vitamin D deficient having parathyroid hyperplasia. In addition, those patients with the highest total 25(OH)D (values greater than 50 ng/mL) had the lowest iPTH median (57% of them with detectable 25(OH)D_2_).

It has been reported that mortality follows a U-shaped curve in relation to the total 25(OH)D concentrations [32–34]. The majority of patients with total 25(OH)D greater than 50 ng/mL had detectable levels of 25(OH)D_2_, indicating that these patients were most likely vitamin D deficient or insufficient and being treated with pharmaceutical vitamin D_2_ (Table 4). This could potentially explain the U-shaped curve regarding increased risk for mortality, fractures and cancer when blood levels exceed 50 ng/mL.

One major limitation of this study is that it is an observational study and thus subject to selection bias, in that there may have been a clinical suspicion that 25(OH)D levels were low. Although the prevalence of vitamin D insufficiency/deficiency may be higher in our study population than the general population, the data are similar to what has been reported by NHANES III and other data sets [19,23,24]. The prevalence of low vitamin D found in our study may be higher than that of the entire population. Because no other biochemical data were analyzed, the study cannot relate other variables such as calcium or phosphate. However, the large number of clinically based results with uniform test methods, provides important insights.

In summary, this study confirms previous reports demonstrating a seasonal, inverse relationship between 25(OH)D_3_, and iPTH [1–6,11,12,14,18]. Our study confirms that this relationship is consistent and reproducible. Novel observations of this study include large variations in the percentage of patients exceeding the upper interval for iPTH or having 25(OH)D_3_ deficiency or insufficiency, the 3.5 week delay between the extremes of 25(OH)D and iPTH, which could also affect interpretation and mapping to the vitamin D, calcium, and parathyroid axis. The month of testing for vitamin D and PTH should be considered and prior clinical studies need reconsideration because of the strong seasonal variation observed in this study. These observations held across three latitudinal regions, both genders, and multiple years, and thus are applicable to general application for patient care. The seasonal relationship must be taken into consideration in the timing and frequency of testing patients and the interpretation of their results.
